# Advanced Heart Failure and End-Stage Heart Failure: Does a Difference Exist

**DOI:** 10.3390/diagnostics9040170

**Published:** 2019-11-01

**Authors:** Paolo Severino, Paul J. Mather, Mariateresa Pucci, Andrea D’Amato, Marco Valerio Mariani, Fabio Infusino, Lucia Ilaria Birtolo, Viviana Maestrini, Massimo Mancone, Francesco Fedele

**Affiliations:** 1 Department of Cardiovascular, Respiratory, Nephrology, Anesthesiology and Geriatric Sciences, Sapienza University of Rome, Viale del Policlinico 155, 00161 Rome, Italy; paolo.severino@uniroma1.it (P.S.); puccimariateresa@gmail.com (M.P.); marcoval.mariani@gmail.com (M.V.M.); fabio.infu@gmail.com (F.I.); ilariabirtolo@gmail.com (L.I.B.); viviana.maestrini@uniroma1.it (V.M.); massimo.mancone@uniroma1.it (M.M.); 2 Department of Medicine, Division of Cardiology University of Pennsylvania, Perelman School of Medicine, 3400 Civic Center Blvd, Philadelphia, PA 19104, USA

**Keywords:** advanced heart failure, end stage heart failure, ventricular assist device, orthotopic heart transplant, HLM classification

## Abstract

Advanced heart failure (AdHF) represents a challenging aspect of heart failure patients. Because of worsening clinical symptoms, high rates of re-hospitalization and mortality, AdHF represents an unstable condition where standard treatments are inadequate and additional interventions must be applied. A heart transplant is considered the optimal therapy for AdHF, but the great problem linked to the scarcity of organs and long waiting lists have led to the use of mechanical circulatory support with ventricular-assist device (VAD) as a destination therapy. VAD placement improves the prognosis, functional status, and quality of life of AdHF patients, with high rates of survival at 1 year, similar to transplant. However, the key element is to select the right patient at the right moment. The complete assessment must include a careful clinical evaluation, but also take into account psychosocial factors that are of crucial importance in the out-of-hospital management. It is important to distinguish between AdHF and end-stage HF, for which advanced therapy interventions would be unreasonable due to severe and irreversible organ damage and, instead, palliative care should be preferred to improve quality of life and relief of suffering. The correct selection of patients represents a great issue to solve, both ethically and economically.

## 1. Introduction

Heart failure (HF) represents a condition in which the heart pump activity cannot guarantee an adequate blood flow to the different organs, compromising their function. 

Globally, HF has a prevalence of greater than 15 million and leads to hundreds of thousands of deaths per year, with mortalities proportionally similar to the infectious epidemics of the Middle Ages. HF represents, in fact, the pandemic of the third millennium. The prevalence of HF in United States (US) and Canada is 1.5%–1.9% of the population, and in Europe it is 1%–2%; hospitalization rates for HF are 1.76%–3.04% in the US and Canada, and 0.32%–3.73% in Europe [1]. HF, unfortunately, is a disease entity that brings both high prevalence and high mortality rate [2]. Among HF classification previously proposed, the New York Heart Association (NYHA) and the American College of Cardiology/American Heart Association (ACC/AHA) are the most used. The NYHA classification [3] divides patients affected by HF into four classes (NYHA I–IV), based on the relationship between the severity of symptoms (dyspnea) and the entity of physical effort necessary to provoke them. Class I includes patients with no symptoms practicing normal physical activity, while patients described as NYHA class II have mild symptoms during usual physical activity, but they are comfortable at rest, with a slight limitation of functional status; class III patients are comfortable only at rest with marked limitation of their functional status, because they experience moderate symptoms with less than normal physical activity. Finally, in class NYHA IV, patients are affected by severe dyspnea also at rest, with, consequently, a severe impairment of their functional status. 

With the aim of supporting and integrating the NYHA classification, the ACC/AHA designed a HF classification based not only on the symptoms but also the cardiovascular risk factors, exercise tolerance and cardiac structural damage among the criteria [4]. In ACC/AHA stage A are included all the patients at high risk of developing HF in the future, without structural or functional heart disorders, while stage B comprises asymptomatic patients with structural heart disease; in stage C are incorporated those patients having previous or current symptoms of HF in a context of structural heart disease, managed by medical treatment; lastly, ACC/AHA stage D includes patients with advanced heart failure (AdHF) who remain symptomatic despite medical support, requiring hospitalization, heart transplant or palliative care.

Among NYHA class IV patients, 89% will eventually die of pump failure. However, a NYHA class IV designation does not signify a uniformly fatal label, without the possibility of intervention. In fact, it is mandatory to go beyond NYHA classes in order to identify and separate patients with AdHF and end-stage HF. Both these conditions are associated with worsening of symptoms, high rates of hospitalization and clinical instability. On one hand, AdHF is a stage where conventional treatments, as such guideline-directed drugs and devices, are insufficient to control patient’s symptoms and advanced therapies are needed and can improve overall survival. On the other hand, in patients with end-stage HF, multiple comorbidities affect the outcomes of advanced therapies and palliation should be the focus of treatment. NYHA classification may not be predictive of outcome, as it does not differentiate those two different stages of HF, both associated to high mortality rates. 

In the US, $47 billion is spent annually on HF [5], of which 53% is on acute inpatient care. In the next few decades the total healthcare expenditure in the US is projected to exceed $1.2 trillion, due to multifactorial etiologies including the aging population and the epidemic of diabetes and obesity that has occurred since the turn of the 21st century [5]. The aging population is a principal driver of the increasing incidence of HF; the prevalence of HF accelerates among those over the age of 75, and this cohort group is currently the fastest growing segment of the global population. Moreover, as the population ages and the incidence of HF hospitalizations increases, more hospital beds will be filled with older patients with HF. Efficient screening and early treatment programs, along with an emphasis on prevention, could significantly reduce healthcare expenditures, thereby freeing up funds for various infrastructures and developments.

The goal of this review manuscript is to better define the prognostic difference between AdHF and end-stage HF. This could be important for choosing the best therapeutic option for patients with HF.

## 2. Advanced Heart Failure (AdHF) and End-Stage Heart Failure: Differences in Clinic, Prognosis and Therapeutic Assessment

Patients with AdHF who have progressive worsening of clinical symptoms, high rates of re-hospitalization, morbidity and mortality, comprise a very important part of the wider HF population. AdHF represents a great issue for both physicians and healthcare systems to solve, both ethically and economically. 

We theorize that differentiating between the entities of AdHF and end-stage HF will be predicated on response to therapies which may include inotropes, vasodilators, ventricular-assistance devices (VAD), and orthotopic heart transplant (OHT).

### 2.1. AdHF and End-Stage HF: Difference in Clinical and Functional Assessment, and Prognosis

AdHF is characterized by the following characteristics: (1) severe symptoms (NYHA class III to IV); (2) episodes with clinical signs of fluid retention and/or peripheral hypoperfusion; (3) objective evidence of severe cardiac dysfunction, shown by at least one of the following: left-ventricular ejection fraction (LVEF) <30%, pseudonormal or restrictive mitral inflow pattern at Doppler-echocardiography; high left- and/or right-ventricular filling pressures; elevated B-type natriuretic peptides; (4) severe impairment of functional capacity demonstrated by either inability to exercise, a 6-min walk test distance <300 m or a peak oxygen uptake <12–14 mL/kg/min; (5) history of >1 HF hospitalisation in the past 6 months; (6) presence of all the previous features despite optimal therapy. This definition identifies a group of patients with compromised quality of life, poor prognosis, and a high risk of clinical events [6]. 

The key to the success as defined by increasing patient life expectancy or quality of life is to define and understand AdHF and end-stage HF because advanced therapy interventions in the end-stage HF patient will inevitably lead to a poor outcome [7].

Current mechanical circulatory support (MCS) guidelines suggest appropriate criteria for implantation of a left-ventricular assist device (LVAD) include an INTERMACS (Interagency Registry for Mechanically Assisted Circulatory Support) 1–3 status, however, outcomes seem to be optimal in INTERMACS 3–4 patients [8]. Reimbursement guidelines do not cover INTERMACS 4 unless the VO2 is <12 mL/kg/min. INTERMACS 3–4 patients (the ambulatory ill with significant dyspnea on exertion and shortness of breath) should be carefully monitored for advanced therapy interventions because their deterioration curves are rapid. More intensive management and surveillance strategies should be considered, which may in some cases reveal poor compliance that could compromise outcomes with any therapy. 

**Figure 1:**
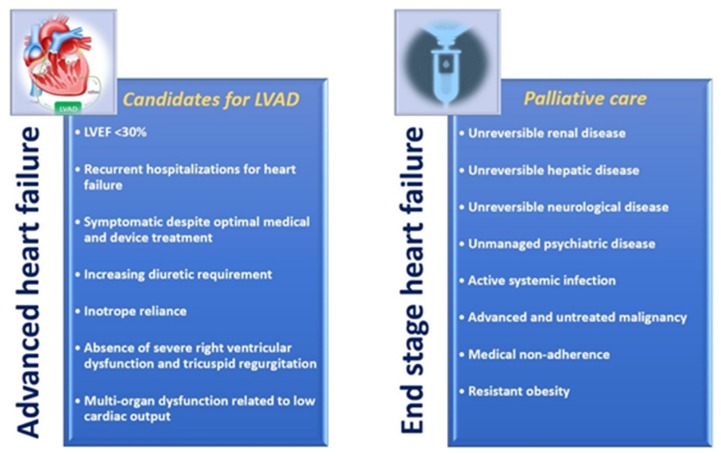
Schematic differences between advanced and end-stage heart failure. LVEF: left-ventricular ejection fraction.

According to recent data, survival in INTERMACS 1 is not as good as in 4 [9], which reinforces the need to treat patients before reaching profile 1. This data emphasizes the need for early recognition and careful selection of patients with AdHF for intervention [10]. The late stage HF patient has severe exercise intolerance, HF wasting syndrome, inotrope dependence, cardiorenal syndrome, and right HF. The clinical delineation between AdHF and end-stage HF is important because intervention is not resurrection [11,12]. VAD are used as a bridge to transplant in patients who are eligible and actively awaiting organs, or as destination therapy in patients unable to receive organ transplant, with a realistic plan for lifelong support. The triggers for VAD implantation in an emergency are as follows: low cardiac index inferior to 2, high filling pressures, maximal inotropic support with or without intra-aortic balloon pump support, declining renal and/or hepatic function that has proved reversible, 0 blood type or body size that predicts a long waiting time for transplant, and intractable arrhythmias. The criteria for MCS as destination therapy are likewise numerous: patients who have chronic AdHF for at least 90 days with a life expectancy of less than 2 years, and are not a candidate for heart transplant; HF symptoms have failed to respond to optimal medical management, including dietary salt restriction, diuretics, digitalis, beta-blockers, and angiotensin-converting enzyme inhibitors (ACE-I) or angiotensin II receptor blockers (ARB) for at least 60 of the last 90 days; LVEF <25% for mechanical reasons; demonstrated functional limitation with a peak oxygen consumption of <12 mL/kg/min or continued need for intravenous inotropic therapy; appropriate body size (in some centers body mass index under 37) to support the VAD implantation (Figure 1). Practically, we can use the acronym “I NEED HELP” [13] to describe patients who are sick, but not moribund, as having AdHF as opposed to end-stage HF. Usually, the former type of patients have presented to hospital or office more than twice in the previous year, they have progressive deterioration in renal function, weight loss without other cause (e.g., cardiac cachexia), and present intolerance to vasodilation that is the worst sign for prognosis since the autonomic system and cardiomyocyte are not responding adequately. They also present with intolerance to beta blockers due to worsening HF or hypotension, with a mortality of 80% in 6 months. They have frequently systolic blood pressure inferior to 90 mm Hg, persistent dyspnea with dressing or bathing requiring rest, inability to walk 150 m on the level ground due to dyspnea or fatigue, a recent need to escalate diuretics to maintain volume status, often reaching daily furosemide equivalent dose >160 mg/d and/or use of supplemental metolazone therapy, progressive decline in serum sodium, usually inferior to 133 mEq/L, and with frequent implantable cardioverter defibrillator (ICD) shocks. Moreover, the presence of myocardial contractile reserve, beyond a value of LVEF <30%, can be helpful to distinguish AdHF and end-stage HF. Indeed, the absence of response to inotropic drugs, suggests an extremely severe cardiomyocyte dysfunction that, along with irreversible organ dysfunction, delineates an end-stage HF not suitable of advanced cardiac therapies. The presence of the aforementioned features suggests an irreversible condition of end-stage HF, for which advanced therapies, such as OHT and VAD implantation, are contraindicated and palliative cares should be pursued. Looking at systemic organ function, AdHF may have hepatic cirrhosis, for congestive hepatopathy or cardiac cirrhosis due to longstanding biventricular failure, excluding biopsies-proven Childs A or B classification, in which advanced mechanical support therapy may not be indicated, except for a heart-liver transplant. Moreover, AdHF patients have intolerance to drugs acting on the renal axis, i.e., ACE-I, ARB or neprylisin inhibitor, due to hypotension and/or worsening renal function, with risk of 20% for mortality in 1 year. Some AdHF patients have neurological impairment such as stroke, but mechanical circulating support therapy is not indicated in patients with ongoing neurological disease or psychiatric disease with body perception alteration. All these AdHF patients may have cardiac cachexia and the need for nutrition added to MCS. Most models focus on patient survival, but some are individuals willing to sacrifice longevity for quality of life [14]. Moreover, since HF is a multiorgan syndrome, assessment of frailty is vitally important in order to assess risk and increase the potential for good response [15,16,17]. 

Regarding prognosis, outcomes of left-ventricular assist device (LVAD) placement in AdHF has improved over the past decade [18,19]; according to recent statistics, there is approximately a 78% chance of living 1 year, and a 68% chance of living 2 years with LVAD placement. HF mortality is increasing despite an overall decline in cardiovascular deaths due to improved survival of patients with diseases leading to HF. Improvements in assessment and management, along with an emphasis on prevention, has impacted greatly on survival from ischemic heart disease and has reduced the co-morbidities triggered by hypertension and diabetes mellitus [20,21,22,23]. These patients are living longer, but ongoing cardiac remodeling is creating a new generation of HF patients. The HF community is focusing on emphasizing aggressive reverse remodeling therapies, such as ACE-I or ARB and β blockers, but the enthusiasm for this emphasis in the general medical community has been uneven. Without using therapies promoting reverse remodeling, 35% of patients with severe HF will die within one year [24,25]. While median survival following diagnosis of HF is 1.7 years for men and 3.2 years for women, around 75% of patients die within 5 years of diagnosis of HF [26]. Moreover, sudden death accounts for 50% of mortality, and in HF it occurs at 6–9 times the rate of the general population. The treatment of HF places a tremendous economic burden on societies. Despite all the therapies, HF morbidity and mortality remain high; 30% to 40% of patients are in NYHA class III or IV. According to a US HF registry, 5-year re-hospitalization and mortality rate are respectively 80.4% and 75.4% [26,27]. In asymptomatic or minimally symptomatic patients, left-ventricular dysfunction therapies are economical and easily tolerated, in general; however, in settings of AdHF, therapeutic interventions will increase in both invasiveness and cost. Thus, early recognition and diagnosis followed by early therapy will lead to lower costs. For many years, hypertension and myocardial infarction were the predominant triggers for HF, but as our diagnostic acumen has delved deeper, the list of triggers has grown. We have come to realize that many co-morbidities lead to a final common pathway of HF. As the number of AdHF cases increases, especially as the population ages, impactful therapies still remain limited. Transplant is still considered the optimal therapy for AdHF [28], however, due to a paucity of organs, mechanical circulatory support with LVADs can be a lifeline bridge [29]. Given the poor health of HF patients, we might ask how long they can reasonably expect to live while waiting for a donor. Nowadays, VAD has become a destination therapy used to manage a patient’s life until they succumb to something else while awaiting a transplant. Around 2000 transplants are performed per year in all the US [30], but as many as 8000 patients with AdHF may be considered for OHT. In patients who are either eligible or not eligible for OHT, VAD is becoming a cardinal alternative for AdHF. One of the keys to maximizing outcomes is early recognition and referral for advanced therapies of AdHF patients. 

### 2.2. How AdHF and End-Stage HF Are Aligned with American College of Cardiology/American Heart Association (ACC/AHA) and New York Heart Association (NYHA) HF Classifications

Regarding the clinical course of HF, when illness progresses and patients come to the hospital for an acute insult, it is not usually possible to rescue them back to where they used to be, and survival will decrease at each decompensation [14]. Unchecked disease progression leads only to palliative options [31,32]. Early recognition and initiation of reverse remodeling therapy may delay the progression of HF and allow for more substantive advanced therapy interventions. If the disease progresses too far, the body may no longer be able to tolerate the stressors of an advanced therapy intervention. It is mandatory to intervene earlier than later so that VADs are not indicated. Therefore, the recognition of an early HF is the best way to prevent its progression and the distinction between the true AdHF and the true end-stage HF patients are fundamental. However, a distinction is not completely reachable using the common classifications as NYHA and ACC/AHA. 

Recently, the Heart Failure Association of the European Society of Cardiology’s (ESC) position paper on AdHF revised criteria for a diagnosis of AdHF [33] from those previously proposed by European and American guidelines [6,34] updating Criterion 2 and Criterion 3. Regarding Criterion 2, it is based on the ESC criteria for cardiac dysfunction giving the same importance to all patients with HF, independent of LVEF. Regarding Criterion 3, it includes HF hospitalization. Malignant arrhythmias have been added as a major cause of acute events. Criterion 3 acknowledges that acute events leading to one or more unplanned visit(s) or hospitalization(s) within 12 months are the hallmark of AdHF, independent of treatment, with emphasis placed on the instability of the clinical course and resource utilization.

AdHF is considered an unstable condition where standard treatment is, by definition, insufficient, and additional interventions must be considered [35,36,37]. Certainly, severe symptoms, such as dyspnea and/or fatigue at rest or with minimal exertion, are keys for identifying AdHF [33]. Episodes of fluid retention are mainly due to non-compliance with diet and medications, but it is also an expression of the progression of cardiomyocyte dysfunction. Another parameter included in AdHF criteria is LVEF less than 30%. However, according to the latest consensus report, a diagnosis of AdHF should not be based solely on LVEF. In fact, it is necessary to highlight that the echocardiographic calculation of LVEF using the Simpson method is relatively unreliable, with well-known intra and interobserver variability [38]. Additionally, LVEF calculation is sensitive to changes in hemodynamic loading conditions, as with severe mitral regurgitation [39,40]. In the management of HF patients, it is more important to focus on ventricular function [41].

Other surrogate markers for function that expand beyond LVEF include functional assessments such as the 6-min walk and the peak oxygen consumption VO2 test. Also, clinical markers such as frequency of admissions to hospitals or acute care settings while on OMT (1 admission within 6 months or 2 within one year) are also known to be indicative of deterioration. 

### 2.3. Revised Criteria for AdHF Diagnostic

AdHF patients have multiorgan dysfunction due to the natural history of the disease. HF is a systemic syndrome with a progressive multiorgan involvement. On one hand, organs such as the kidney, lungs, liver and brain are affected by chronic low cardiac output and inflammatory status triggered by HF, so that the worse the cardiac performance, the worse the multiorgan failure. On the other hand, chronic kidney disease, vascular encephalopathy, vascular dementia, anemia, lung and hepatic failures worsen patients’ global health and prognosis. Moreover, these comorbidities affect cardiac performance (for instance, fluid overload in renal failure and right HF in the presence of pulmonary arterial hypertension) and worsen prognosis. The presence of severe non-cardiac comorbidity is strongly associated to worsening of patients’ global status and predicts an irreversible, end-stage HF syndrome. In this view, the capability of staging HF beyond cardiac performance and symptoms is pivotal to distinguish AdHF and end-stage HF, and can be accomplished using HLM nosology [42,43]. In fact, we recently proposed a new staging system for HF similar to the TNM classification used in oncology, named HLM, in order to identify the systemic involvement of the disease. In HLM classification, “H” is heart, analogous to the ‘T” in TNM, “L” is for lungs that are anatomically and functionally correspondent to lympho-nodes of the heart; similarly to the concept of metastasis used in oncology, the implication of other organs is identified with “M” (the kidney, the liver, the central nervous system and the hematopoietic system). Regarding cardiac damage in HF, it progresses from an initial stage of impaired systolic or diastolic left-ventricular function, without any structural injury, based upon calculations of wall thickness, cavity diameters, wall kinesis and valves (H1), to a structural cardiac damage, i.e., left-ventricle concentric wall hypertrophy, abnormal kinesis of left-ventricle walls, moderate to severe valvular disease (H2), to left-ventricle dilation or remodelling (H3), to an advanced stage of biventricular dysfunction (H4). Regarding L parameter, it ranges from the absence of any lung involvement (L0), to hemodynamic (L1) or clinical pulmonary congestion (L2), to the advanced cardiac lung (L3) with arterialization of pulmonary vasculature. Finally, the last parameter of HLM identifies any malfunction (M) of one or more peripheral organs, such as the kidney, liver, brain, and hematopoietic system, to the cardiac cachexia, which is the expression of multiorgan failure. The HLM classification was influenced by the key elements of TNM staging: simplicity, clinical usefulness, efficacy for planning a therapeutic strategy, and the ability to determine patient prognosis. HLM classification seems to be easily applied in the real world and presents a valuable tool for balancing economic resources with the clinical complexity of patients [44].

Moreover, in this setting, evaluating both clinical, instrumental and laboratory data for cardiac, pulmonary and systemic dysfunction, we might better identify patients with true AdHF or end-stage HF in order to start the best therapy in terms of ethical and economic appropriateness. In view of HF patients, traditional therapies are indicated for the initial stage of HLM, whereas more expensive second-tier therapies may be required in more compromised stages. If systemic organs are involved, like “metastasis”, cardio-, nephro-, and hepato-protective therapies are indicated, justifying the increased costs of therapy in proportion to the obtained benefits [45,46,47,48]. Finally, in assessing the true end-stage patients, the aim should be to pursue the most appropriate, therapy targeting the quality more than the quantity of life.

The approach of HLM classification aims to outweigh the cardiocentric vision of HF that so far seems to be unable to discriminate the true AdHF and the end-stage HF patient. A multicenter data analysis of outcomes on LVAD candidates is ongoing in Italy and in United States, and the results will confirm the validity of HLM which seems to be more accurate than other classifications in terms of risk stratification for hospitalization for HF and for cardiac death at 12 months’ follow-up [49,50].

## 3. Conclusions

HF represents one of the main cause of hospitalization and death and it has also a great impact on public health spending. For these reasons, identifying precociously patients affected by HF and classifying the stage of their disease may be very important to establish a correct therapy to improve and lengthen their life, together reducing public health spending. In particular we focused on two stages of HF: AdHF, an unstable condition in which a standard therapy is inadequate but other advanced approaches such as OHT and the VAD application may be decisive for patient survival, and end-stage HF a condition in which a diffuse organs damage is already established and the patient could be referred only to palliative care. Distinguishing patients with AdHF from end-stage HF represents the most important challenge at the moment. The difference between the two conditions is not only clinical but also prognostic and therapeutic. Our HLM classification may be useful to better identify AdHF patients for which an advanced therapy is reasonable.

## References

[1] Ponikowski P., Anker S.D., Al Habib K.F., Cowie M.R., Force T.L., Hu S., Jaarsma T., Krum H., Rastogi V., Rohde L.E. (2014). Heart failure: Preventing disease and death worldwide. ESC Heart Fail..

[2] Hjalmarson A., Goldstein S., Fagerberg B., Wedel H., Waagstein F., Kjekshus J., Wikstrand J., Westergren G., Thimell M., El Allaf D. (1999). Effect of metoprolol CR/XL in chronic heart failure: Metoprolol CR/XL Randomised Intervention Trial in Congestive Heart Failure (MERIT-HF). Lancet.

[3] Dolgin M., The Criteria Committee of the New York Heart Association (1994). Functional Capacity and Objective Assessment. Nomenclature and Criteria for Diagnosis of Diseases of the Heart and Great Vessels.

[4] Yancy C.W., Jessup M., Bozkurt B., Butler J., Casey D.E., Drazner M.H., Fonarow G.C., Geraci S.A., Horwich T., Januzzi J.L. (2017). 2017 ACC/AHA/HFSA Focused Update of the 2013 ACCF/AHA Guideline for the Management of Heart Failure. J. Am. Coll. Cardiol..

[5] Lloyd-Jones D., Adams R., Carnethon M., De Simone G., Ferguson T.B., Flegal K., Ford E., Furie K., Go A., Greenlund K. (2009). Heart disease and stroke statistics-2009 update: A report from the American Heart Association Statistics Committee and Stroke Statistics Subcommittee. Circulation.

[6] Metra M., Ponikowski P., Dickstein K., McMurray J.J., Gavazzi A., Bergh C.H., Fraser A.G., Jaarsma T., Pitsis A., Mohacsi P. (2007). Advanced chronic heart failure: A position statement from the study group on advanced heart failure of the Heart Failure Association of the European Society of Cardiology. Eur. J. Heart Fail..

[7] Van der Meer P., Gaggin H.K., Dec G.W. (2019). ACC/AHA Versus ESC Guidelines on Heart Failure: JACC Guideline Comparison. J. Am. Coll. Cardiol..

[8] Stewart G.C., Stevenson L.W. (2011). Keeping left ventricular assist device acceleration on track. Circulation.

[9] Kirklin J.K., Pagani F.D., Kormos R.L., Stevenson L.W., Blume E.D., Myers S.L., Miller M.A., Baldwin J.T., Young J.B., Naftel D.C. (2017). Eighth annual INTERMACS report: Special focus on framing the impact of adverse events. J. Heart Lung Transplant..

[10] Wilson S.R., Mudge G.H., Stewart G.C., Givertz M.M. (2009). Evaluation for a ventricular assist device: Selecting the appropriate candidate. Circulation.

[11] Fedele F., Severino P., Bruno N., Stio R., Caira C., D’Ambrosi A., Brasolin B., Ohanyan V., Mancone M. (2013). Role of ion channels in coronary microcirculation: A review of the literature. Future Cardiol..

[12] Fedele F., Mancone M., Chilian W.M., Severino P., Canali E., Logan S., De Marchis M.L., Volterrani M., Palmirotta R., Guadagni F. (2013). Role of genetic polymorphisms of ion channels in the pathophysiology of coronary microvascular dysfunction and ischemic heart disease. Basic Res. Cardiol..

[13] Baumwol J. (2017). I Need Help—A mnemonic to aid timely referral in advanced heart failure. J. Heart Lung Transplant..

[14] Allen L.A., Stevenson L.W., Grady K.L., Goldstein N.E., Matlock D.D., Arnold R.M., Cook N.R., Felker G.M., Francis G.S., Hauptman P.J. (2012). Decision making in advanced heart failure: A scientific statement from the American Heart Association. Circulation.

[15] Flint K.M., Matlock D.D., Lindenfeld J., Allen L.A. (2012). Frailty and the selection of patients for destination therapy left ventricular assist device. Circ. Heart Fail..

[16] Severino P., Netti L., Mariani M.V., Maraone A., D’Amato A., Scarpati R., Infusino F., Pucci M., Lavalle C., Maestrini V. (2019). Prevention of Cardiovascular Disease: Screening for Magnesium Deficiency. Cardiol. Res. Pract..

[17] Severino P., D’Amato A., Pucci M., Mariani M.V., Netti L., Infusino F., Mancone M., Fedele F. (2019). Myocardial Ischemia in Women When Genetic Susceptibility Matters. J. Mol. Genet. Med..

[18] Miller R.J.H., Teuteberg J.J., Hunt S.A. (2019). Innovations in Ventricular Assist Devices for End-Stage Heart Failure. Annu. Rev. Med..

[19] Miller L.W., Rogers J.G. (2018). Evolution of Left Ventricular Assist Device Therapy for Advanced Heart Failure: A Review. JAMA Cardiol..

[20] De Freitas Campos Guimarães L., Urena M., Wijeysundera H.C., Munoz-Garcia A., Serra V., Benitez L.M., Auffret V., Cheema A.N., Amat-Santos I.J., Fisher Q. (2018). Long-term outcomes after transcatheter aortic valve-in-valve replacement. Circ. Cardiovasc. Interv..

[21] Meier B. (2017). His master’s art, Andreas Grüntzig’s approach to performing and teaching coronary angioplasty. EuroIntervention.

[22] Basoli A., Cametti C., Satriani F.G., Mariani P., Severino P. (2012). Hemocompatibility of stent materials: Alterations in electrical parameters of erythrocyte membranes. Vasc. Health Risk Manag..

[23] Severino P., D’Amato A., Netti L., Pucci M., De Marchis M., Palmirotta R., Volterrani M., Mancone M., Fedele F. (2018). Diabetes mellitus and ischemic heart disease: The role of ion channels. Int. J. Mol. Sci..

[24] Adams K.F., Baughman K.L., Dec W.G., Elkayam U., Forker A.D., Gheorghiade M., Hermann D., Konstam M.A., Liu P., Massie B.M. (2000). HFSA guidelines for management of patients with heart failure caused by left ventricular systolic dysfunction-pharmacological approaches. Pharmacotherapy.

[25] Ho K.K., Pinsky J.L., Kannel W.B., Levy D. (1993). The epidemiology of heart failure: The Framingham Study. J. Am. Coll. Cardiol..

[26] Shah K.S., Xu H., Matsouaka R.A., Heidenreich P.A., Hernandez A.F., Devore A.D., Yancy C.W., Fonarow G.C. (2017). Heart failure with preserved, borderline, and reduced ejection fraction: 5-year outcomes. J. Am. Coll. Cardiol..

[27] Lloyd-Jones D., Adams R.J., Brown T.M., Carnethon M., Dai S., De Simone G., Ferguson T.B., Ford E., Furie K., Gillespie C. (2010). Heart Disease and Stroke Statistics-2010: A report from the American Heart Association. Circulation.

[28] Levine A., Gupta C.A., Gass A. (2019). Advanced Heart Failure Management and Transplantation. Cardiol. Clin..

[29] Miller L., Birks E., Guglin M., Lamba H., Frazier O.H. (2019). Use of Ventricular Assist Devices and Heart Transplantation for Advanced Heart Failure. Circ. Res..

[30] Everly M.J. (2008). Cardiac transplantation in the United States: An analysis of the UNOS registry. Clin. Transpl..

[31] Lowey S.E. (2018). Palliative Care in the Management of Patients with Advanced Heart Failure. Adv. Exp. Med. Biol..

[32] Martens P., Vercammen J., Ceyssens W., Jacobs L., Luwel E., Van Aerde H., Potargent P., Renaers M., Dupont M., Mullens W. (2018). Effects of intravenous home dobutamine in palliative end-stage heart failure on quality of life, heart failure hospitalization, and cost expenditure. ESC Heart Fail..

[33] Crespo-Leiro M.G., Metra M., Lund L.H., Milicic D., Costanzo M.R., Filippatos G., Gustafsson F., Tsui S., Barge-Caballero E., De Jonge N. (2018). Advanced heart failure: A position statement of the Heart Failure Association of the European Society of Cardiology. Eur. J. Heart Fail..

[34] Yancy C.W., Jessup M., Bozkurt B., Butler J., Casey D.E., Drazner M.H., Fonarow G.C., Geraci S.A., Horwich T., Januzzi J.L. (2013). 2013 ACCF/AHA guideline for the management of heart failure: A report of the American College of Cardiology Foundation/American Heart Association Task Force on practice guidelines. J. Am. Coll. Cardiol..

[35] Severino P., D’Amato A., Netti L., Pucci M., Infusino F., Maestrini V., Mancone M., Fedele F. (2019). Myocardial Ischemia and Diabetes Mellitus: Role of Oxidative Stress in the Connection between Cardiac Metabolism and Coronary Blood Flow. J. Diabetes Res..

[36] Severino P., Mariani M.V., Maraone A., Piro A., Ceccacci A., Tarsitani L., Maestrini V., Mancone M., Lavalle C., Pasquini M. (2019). Triggers for Atrial Fibrillation: The Role of Anxiety. Cardiol. Res. Pract..

[37] Severino P., Maestrini V., Mariani M.V., Birtolo L.I., Scarpati R., Mancone M., Fedele F. (2019). Structural and myocardial dysfunction in heart failure beyond ejection fraction. Heart Fail. Rev..

[38] Wood P.W., Choy J.B., Nanda N.C., Becher H. (2014). Left ventricular ejection fraction and volumes: It depends on the imaging method. Echocardiography.

[39] Gaasch W.H., Meyer T.E. (2008). Left ventricular response to mitral regurgitation: Implications for management. Circulation.

[40] Berko B., Gaasch W.H., Tanigawa N., Smith D., Craige E. (1987). Disparity between ejection and end-systolic indexes of left ventricular contractility in mitral regurgitation. Circulation.

[41] Fedele F., Mancone M., Adamo F., Severino P. (2017). Heart failure with preserved, mid-range, and reduced ejection fraction: The misleading definition of the new guidelines. Cardiol. Rev..

[42] Fedele F., Gatto M.C., D’Ambrosi A., Mancone M. (2013). TNM-like classification: A new proposed method for heart failure staging. Sci. World, J..

[43] Fedele F., Severino P., Calcagno S., Mancone M. (2014). Heart failure: TNM-like classification. J. Am. Coll. Cardiol..

[44] Maestrini V., Birtolo L.I., Cimino S., Severino P., Mancone M., Francone M., Banypersad S.M., Ventriglia F., Tritapepe L., Miraldi F. (2019). Giant right atrium and subvalvular pulmonary stenosis: A case report of an interesting combination. Echocardiography.

[45] Nieminen M.S., Buerke M., Parissis J., Ben-Gal T., Pollesello P., Kivikko M., Karavidas A., Severino P., Comín-Colet J., Wikström G. (2015). Pharmaco-economics of levosimendan in cardiology: A European perspective. Int. J. Cardiol..

[46] Gold M.R., Padhiar A., Mealing S., Sidhu M.K., Tsintzos S.I., Abraham W.T. (2017). Economic value and cost-effectiveness of cardiac resynchronization therapy among patients with mild heart failure: Projections from the REVERSE Long-Term Follow-Up. J. Am. Coll. Cardiol. HF.

[47] Sandhu A.T., Ollendorf D.A., Chapman R.H., Pearson S.D., Heidenreich P.A. (2016). Cost-effectiveness of Sacubitril-Valsartan in patients with heart failure with reduced ejection fraction. Ann. Intern. Med..

[48] Ollendorf D., Sandhu A.T., Pearson S.D. (2016). Sacubitril-Valsartan for the treatment of heart failure effectiveness and value. JAMA Intern. Med..

[49] Severino P., Scarpati R., Pucci M., Alfarano M., Infusino F., Cimino S., Calcagno S., Alunni Fegatelli D., Maestrini V., Vestri A. (2019). Prognostic role of TNM-like classification for heart failure at 12 months of follow-up: Comparison with others nosologies. J. Am. Coll. CardioL..

[50] Severino P., Mariani M.V., Fedele F. (2019). Futility in cardiology: The need for a change in perspectives. Eur. J. Heart Fail..

